# A computational framework for testing hypotheses of the minimal mechanical requirements for cell aggregation using early annual killifish embryogenesis as a model

**DOI:** 10.3389/fcell.2023.959611

**Published:** 2023-03-20

**Authors:** Ignacio Montenegro-Rojas, Guillermo Yañez, Emily Skog, Oscar Guerrero-Calvo, Martin Andaur-Lobos, Luca Dolfi, Alessandro Cellerino, Mauricio Cerda, Miguel L. Concha, Cristina Bertocchi, Nicolás O. Rojas, Andrea Ravasio, Timothy J. Rudge

**Affiliations:** ^1^ Laboratory for Mechanobiology of Transforming Systems, Institute for Biological and Medical Engineering, Schools of Engineering, Medicine and Biological Sciences. Pontificia Universidad Católica de Chile, Santiago, Chile; ^2^ Institute for Biological and Medical Engineering, Schools of Engineering, Medicine and Biological Sciences. Pontificia Universidad Católica de Chile, Santiago, Chile; ^3^ Interdisciplinary Computing and Complex Biosystems (ICOS) Research Group, School of Computing, Newcastle University, Newcastle upon Tyne, United Kingdom; ^4^ Max Planck Institute for Biology of Ageing, Cologne, Germany; ^5^ Center for Anatomy and Cell Biology, Medical University of Vienna, Vienna, Austria; ^6^ BIO@SNS, Scuola Normale Superiore, Pisa, Italy; ^7^ Leibniz Institute on Aging - Fritz Lipmann Institute, Jena, Germany; ^8^ Integrative Biology Program, Institute of Biomedical Sciences, Facultad de Medicina. Universidad de Chile, Santiago, Chile; ^9^ Biomedical Neuroscience Institute, Santiago, Chile; ^10^ Center for Medical Informatics and Telemedicine, Facultad de Medicina, Universidad de Chile, Santiago, Chile; ^11^ Center for Geroscience, Brain Health and Metabolism, Santiago, Chile; ^12^ Laboratory for Molecular Mechanics of Cell Adhesion, Department of Physiology Pontificia Universidad Católica de Chile, Santiago, Chile; ^13^ Graduate School of Engineering Science, Osaka University, Osaka, Japan

**Keywords:** multicellularity, mechanics, biophysics, killifish, adhesion, modeling

## Abstract

**Introduction:** Deciphering the biological and physical requirements for the outset of multicellularity is limited to few experimental models. The early embryonic development of annual killifish represents an almost unique opportunity to investigate *de novo* cellular aggregation in a vertebrate model. As an adaptation to seasonal drought, annual killifish employs a unique developmental pattern in which embryogenesis occurs only after undifferentiated embryonic cells have completed epiboly and dispersed in low density on the egg surface. Therefore, the first stage of embryogenesis requires the congregation of embryonic cells at one pole of the egg to form a single aggregate that later gives rise to the embryo proper. This unique process presents an opportunity to dissect the self-organizing principles involved in early organization of embryonic stem cells. Indeed, the physical and biological processes required to form the aggregate of embryonic cells are currently unknown.

**Methods:** Here, we developed an *in silico*, agent-based biophysical model that allows testing how cell-specific and environmental properties could determine the aggregation dynamics of early Killifish embryogenesis. In a forward engineering approach, we then proceeded to test two hypotheses for cell aggregation (cell-autonomous and a simple taxis model) as a proof of concept of modeling feasibility. In a first approach (cell autonomous system), we considered how intrinsic biophysical properties of the cells such as motility, polarity, density, and the interplay between cell adhesion and contact inhibition of locomotion drive cell aggregation into self-organized clusters. Second, we included guidance of cell migration through a simple taxis mechanism to resemble the activity of an organizing center found in several developmental models.

**Results:** Our numerical simulations showed that random migration combined with low cell-cell adhesion is sufficient to maintain cells in dispersion and that aggregation can indeed arise spontaneously under a limited set of conditions, but, without environmental guidance, the dynamics and resulting structures do not recapitulate *in vivo* observations.

**Discussion: **Thus, an environmental guidance cue seems to be required for correct execution of early aggregation in early killifish development. However, the nature of this cue (e.g., chemical or mechanical) can only be determined experimentally. Our model provides a predictive tool that could be used to better characterize the process and, importantly, to design informed experimental strategies.

## 1 Introduction

**FIGURE 1:**
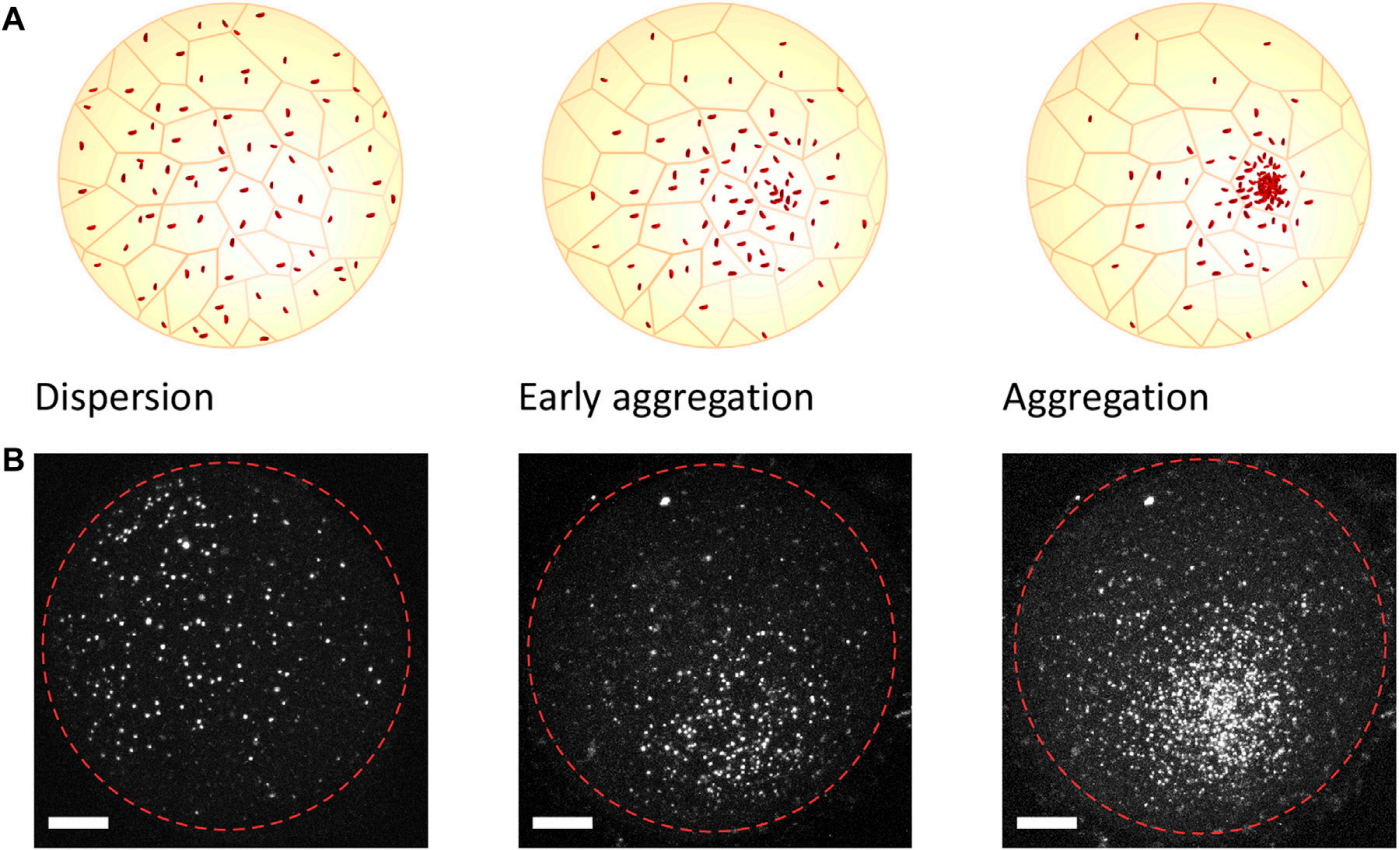
Stages of the early development of annual killifish as recorded *in vivo*. Cartoon representation **(A)** and confocal images of embryonic cell nuclei **(B)** stained using the FUCCI construct, illustrating the stages of the early development of killifish embryos. Following the dispersive state (left), the cells move directionally toward a pole of the embryo (center) and form an aggregate (right). The dashed red circles indicate the approximate outline of the embryo. The time between the stages varies between several hours to a few days, depending on the environmental condition and specific killifish species (Dolfi et al., 2014; Márquez et al., 2019). Scale bar, 200 μm.

Annual killifish have a unique early developmental pattern that differs from most teleost species. Unlike non-annual species, for whom most morphogenetic movements are concomitant, annual killifish have separated epiboly from embryo formation, resulting in an initial phase of cell dispersal that is followed by a process of cell aggregation, with embryonic cells occurring through active cell migration in the confined space between the enveloping layer (EVL) and the yolk syncytial layer (YSL) (Figure 1) (Dolfi et al., 2014; Concha and Reig, 2022). The embryonic cells remain undifferentiated, with stem-like properties during epiboly and until the end of the dispersion phase, when the cells start to aggregate at one pole of the embryo and initiate the genetic and morphogenetic processes leading to the formation of germ layers and the establishment of the embryonic axis (Wourms, 1972; Pereiro et al., 2017; Márquez et al., 2019) (Figure 1). These processes, which span between several hours and a few days depending on the specific killifish species and the environmental conditions (Dolfi et al., 2014; Márquez et al., 2019), occur in the context of a nearly spherical egg (about 1 mm diameter) and can be easily visualized in a living animal, as eggs are optically transparent and develop outside the mother (Wourms, 1972; Pereiro et al., 2017; Reig et al., 2017; Concha and Reig, 2022). The dispersion phase is characterized by a random walk of embryonic cells moving at a very low density (Márquez et al., 2019), while the cellular processes and morphogenetic mechanisms that form the aggregate are still unknown. It has been proposed that self-organizing processes may break the initial symmetry of the embryo and initiate the aggregation process (Pereiro et al., 2017; Abitua et al., 2021), since the molecular signals involved in embryo formation are apparently non-polarized during the stages prior to the aggregate formation. However, it cannot be ruled out that an organizing center, possibly located in extraembryonic structures, provides the signals that initiate the aggregate formation (Pereiro et al., 2017) as has been shown in other non-annual teleost species (Concha and Reig, 2022).

*In silico* modeling proved to be a powerful tool to accurately capture the essential features of various biological systems, such as wound healing (Ravasio et al., 2015a), tissue expansion (Ravasio et al., 2015b), cancer invasion (Stichel et al., 2017), and embryonic development (CONTE et al., 2008; Cai et al., 2016; Pereiro et al., 2017; Stepien et al., 2019). Thus, it has been proposed that they could be used as predictive tools to design informed experimental strategies (Kabla, 2012; Phillips, 2015). Here, we used an *in silico* model to understand the mechanical requirements for killifish cells to 1) remain in a dispersed state and 2) aggregate at the embryo pole to initiate embryogenesis. In our model, motile cells are represented by three-dimensional self-propelled particle spheres, which is a 3D framework commonly used to model collective cell dynamics (Belmonte et al., 2008; Henkes et al., 2011; Sepúlveda et al., 2013; Méhes and Vicsek, 2014; Tarle et al., 2015). This approach has been extended to incorporate biologically relevant interactions such as cell–substrate friction, intercellular and cell–substrate adhesions (Kanchanawong et al., 2010; Bertocchi et al., 2017), and contact inhibition of locomotion (CIL) (Abercrombie and Heaysman, 1954; Roycroft and Mayor, 2016). When generalized, this model can exhibit diverse dynamic states, such as gas phases, polar liquids, and 3D aggregates, depending on the parameter explored (Bray, 1993; Mladek et al., 2006; Moreno and Likos, 2007; Redner et al., 2013). Although these states were experimentally observed at high cell densities, it is an open question as to whether such mechanisms could account for the aggregation behavior observed at low cell densities found in the early stages of killifish development. Furthermore, to date, models have not considered the specific geometry of this process, such as the cells moving in confinement and on curved surfaces with spherical topology. Typically, these studies use periodic boundary conditions on a plane, giving a toroidal topology (Bellomo et al., 2015). Our model incorporates realistic conditions in terms of cell density, geometrical and mechanical properties of the EVL and the YSL, and their effect on the dynamics of embryonic cells. Thus, the *in silico* investigation presented aims to provide a flexible framework to model the early teleost development, which can help predict the minimal mechanical requirements for cell aggregation under the specific conditions of annual killifish early development. As the *in vivo* system is poorly understood and presents various intrinsic experimental challenges (e.g., coriaceous chorion), our forward engineering approach can provide useful information, enabling an informed experimental investigation of the biological system.

## 2 Results

**FIGURE 2:**
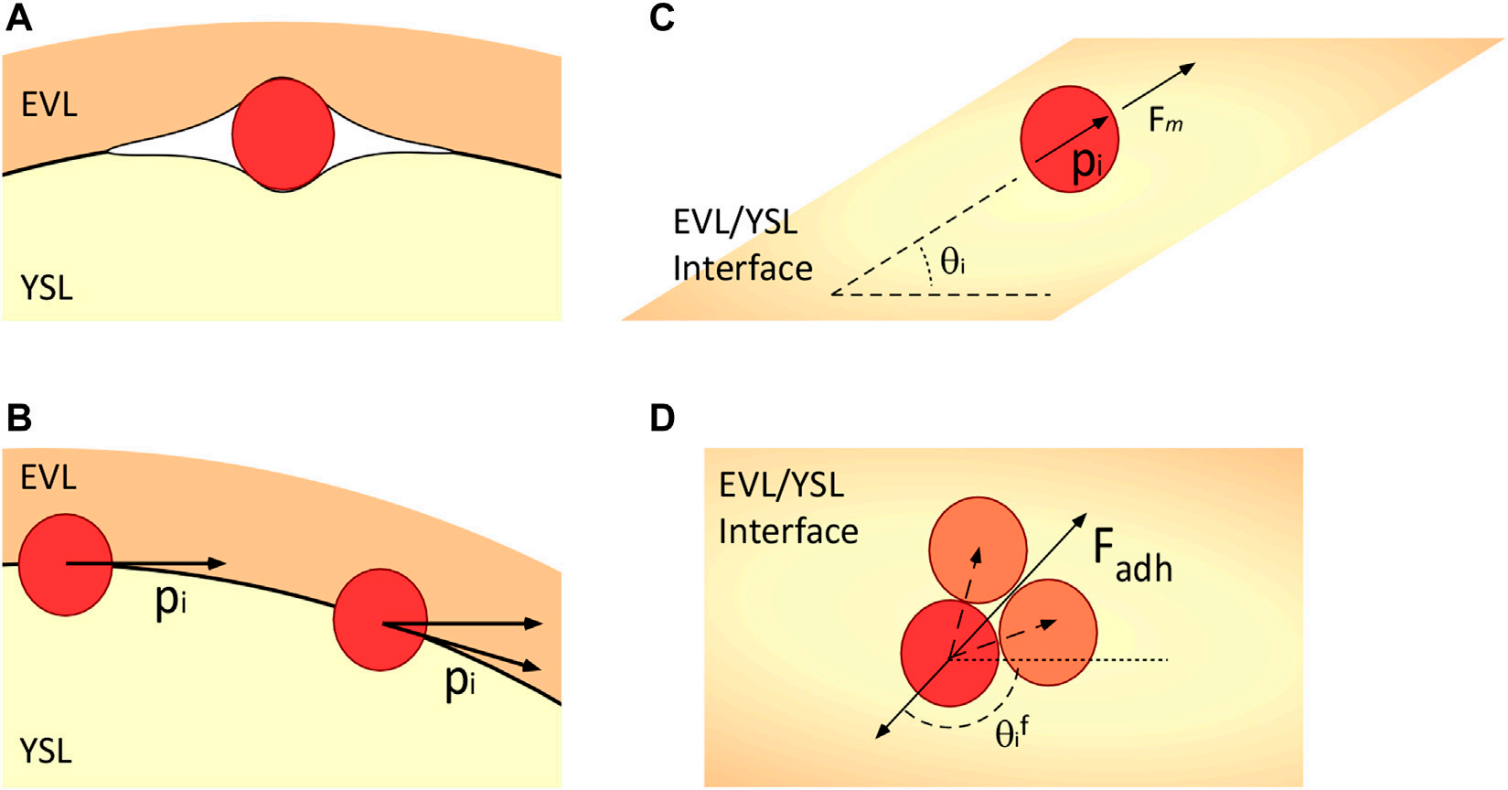
Schematic representation of the model’s major components. **(A)** Embryonic cells move between the EVL and the YSL, which are roughly spherical. They are subject to forces due to the deformation of the adjacent cell layers. **(B)** Polarity of the cells is constrained to the tangential plane as they move. **(C)** Contact inhibition of locomotion repolarizes cells away from the average position of their neighbors, and adhesion forces attract them to their neighbors. **(D)** Angle with respect to the average neighbor position. The dashed vectors indicate the directions to the neighbors of the red cell, the bottom solid arrow indicates the reference direction, and $\theta_{i}^{f}$ is the angle to which the cells repolarize due to CIL. The direction of the adhesion force is shown for reference.

During the early stages of killifish embryo development, undifferentiated stem cells move tangentially between the inner surface of the epithelial enveloping cell layer and the yolk syncytial layer (Figure 2A). The system is modeled here in two distinct ways: a cell-autonomous system that includes mechanisms that are intrinsic to the cells and the same cells that are under the influence of guidance from the environment toward an organizing center.

### 2.1 Cell-autonomous system

We present, here, a variation of the model proposed by Smeets et al. (2016) (Basan et al., 2013) for autonomous motile cells, extended to three dimensions and including the physical and geometrical constraints imposed by the EVL and the YSL, where the cells migrate tangentially to the surface of the YSL (Figure 2B).

#### 2.1.1 Equations of motion

The cells have an intrinsic motile force, $F_{m}$ , that drives them forward along their direction of polarity ${\hat{p}}_{i}$ (Figure 2C). The cells are subject to viscous forces from the substrate with the coefficient $\gamma_{s}$ and from other cells $F_{ij}^{cc}$ . The equation of motion is as follows:

$$F_{m}{\hat{p}}_{i}=\gamma_{s}{\dot{x}}_{i}+\underset{j}{\sum }F_{ij}^{cc}{\hat{n}}_{ij}$$ (1)

with the left-hand term and the motile force with direction ${\hat{p}}_{i}$ , where $\gamma_{s}$ is the substrate viscosity and $F_{ij}^{cc}$ is the force between the cells i and j , acting in the normal direction to their surface ${\hat{n}}_{ij}$ at the point of contact. The normal direction at the point of contact in the case of the two spheres is simply the direction between their two centers so that:

$${\hat{n}}_{ij}=\frac{\left({x_{i}-x}_{j}\right)}{d_{ij}},$$ (2)

with $d_{ij}=\left|{x_{i}-x}_{j}\right|$ being the distance between cells. The force $F_{ij}^{cc}$ is defined in terms of $d_{ij}$ .

#### 2.1.2 Cell–cell adhesion and repulsion

The adhesion and repulsion between cells may play a significant role in the formation of aggregates. These forces are determined by the interaction between the cell–cell adhesion energy $W_{c}$ and the cell–substrate adhesion energy $W_{s}$ , such that the intercellular force is given by (Smeets et al., 2016):

$$F_{ij}^{cc}=\frac{2}{R}\left[W_{s}-\frac{W_{s}+W_{c}}{R}\left(d_{ij}-R\right)\right],$$ (3)

for pairs of cells *i* and *j* in contact ( $d_{ij}<2R$ ) with the cell radius R . Here, $F_{ij}^{cc}=0$ when $d_{ij}\geq 2R$ , as the cells are not in contact. We model the EVL and the YSL as single large spherical cells of radius $R_{E}$ centered on the origin:

$$F_{i}^{YSL}=\frac{2}{R}\left[W_{s}-\frac{W_{s}+W_{c}}{R}\left(\left|x_{i}\right|-R_{E}-R\right)\right],$$

which is the force applied to cell *i* by the YSL when $\left|x_{i}\right|-R_{E}<R$ ( $F_{i}^{YSL}=0$ otherwise), and

$$F_{i}^{EVL}=\frac{2}{R}\left[W_{s}-\frac{W_{s}+W_{c}}{R}\left(R_{E}-\left|x_{i}\right|-R\right)\right],$$

which is the force applied to cell *i* by the EVL when $R_{E}-\left|x_{i}\right|<R$ ( $F_{i}^{EVL}=0$ otherwise). In this way, the cells experience forces that tend to maintain them on a sphere of radius $R_{E}$ . It should be noted that unlike in the work of Smeets et al. (2016), there is no cut-off of intercellular forces when the cells are closer than *R*, and no cells are removed from the simulation to model multiple layers. This is not necessary since our model is fully three-dimensional, and due to the spherical geometry, cells are naturally forced outward to form multilayered aggregates, which can be observed under large adhesive energies. Since the dynamics of the system depend upon the relation between cell–cell and cell–substrate energies rather than their absolute strengths, from here on, we use $W_{s}=1$ and simply vary $W_{c}$ .

#### 2.1.3 Repolarization and rotational diffusion

In our model, the cell polarity vector ${\hat{p}}_{i}$ is constrained to the tangential plane between the EVL and the YSL, and the motile force is as follows:

$$F_{m}=F_{m}{\hat{p}}_{i},$$ (4)

where ${\hat{p}}_{i}={\left(\cos\theta_{i},\sin\theta_{i}\right)}^{T}$ , and $F_{m}$ is the magnitude of the motile force. We used the model by Smeets et al. (2016) for the repolarization of the direction vector of the cell ${\hat{p}}_{i}$ , which has angle $\theta_{i}$ in the tangential plane (Figure 2C):

$${\dot{\theta }}_{i}=-f_{pol}\left(\theta_{i}-\theta_{i}^{*}\right)+\xi \sqrt{2D_{r}},$$ (5)

where $\theta_{i}^{*}$ is the target or desired direction of the cell, $f_{pol}$ is the rate of repolarization, $D_{r}$ is the rate of angular diffusion, and ξ is a Gaussian noise process. We may define any formulation for the desired direction depending on which type of repolarization process we wish to consider. This equation can be normalized by giving a single dimensionless parameter, $\psi =f_{pol}/2D_{r}$ . In the following, we fix $D_{r}$ at unity.

#### 2.1.4 Contact inhibition of locomotion

Contact inhibition of locomotion (CIL), which works as a repulsion interaction causing cells to steer away from each other, is another important determinant of cell migration, which we wish to test in our model. For CIL, we model the repolarization direction, $\theta_{i}^{*}=\theta_{i}^{f}$ , as the direction pointing away from the average position of each cell’s neighbors (contacting cells—Figure 2D), so if the average position of the neighboring cells is

$${\bar{x}}_{i}=\underset{j}{\sum }x_{j},$$

for the neighboring cells j, the desired direction vector in the tangent plane with normal ${\hat{n}}_{i}$ is

$$v_{i}^{f}=\left({\bar{x}}_{i}-x_{i}\right)-\left[\left({\bar{x}}_{i}-x_{i}\right)\cdot {\hat{n}}_{i}\right]{\hat{n}}_{i}.$$

Thus, the cells will turn away from contacting cells at a rate of $f_{pol}=f_{cil}$ , so that

$${\dot{\theta }}_{i}=-f_{cil}\times \arccos\left(\frac{v_{i}^{f}∙{\hat{p}}_{i}}{\left|v_{i}^{f}\right|}\right)+\sqrt{2D_{r}}\xi .$$ (6)

The system can again be normalized by the dimensionless parameter $\psi =f_{cil}/2D_{r}$ since $f_{pol}=f_{cil}$ .

#### 2.1.5 Numerical simulations

**FIGURE 3:**
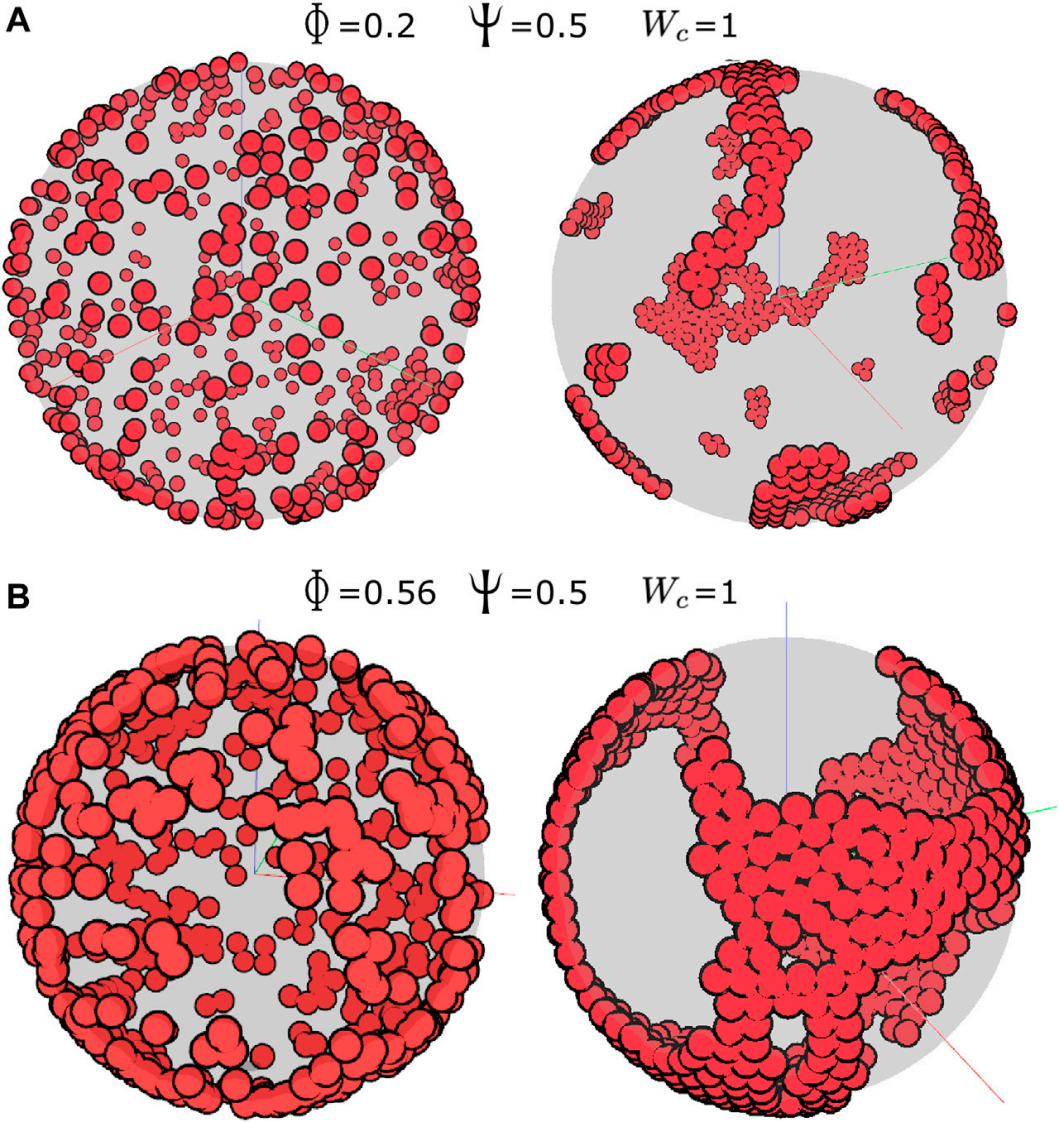
Aggregation in the cell-autonomous system. **(A)** Cell density ϕ=0.2 (500 cells on a sphere of radius 25), ψ=0.5 , and $W_{c}=1.$ **(B)** Cell density ϕ=0.56 (500 cells on a sphere of radius 15), ψ=0.5 , and $W_{c}=1$ . The images on the left show the initial conditions, and the images on the right show the simulations after 500 time steps. The YSL is drawn as a transparent gray sphere; hence, the cells on the far side appear darker. The three-dimensional perspective means that these cells also appear smaller.

This model can be solved numerically (see Methods) for a range of parameters, providing a powerful tool to simulate developmental processes occurring in a realistic geometrical setting. Using the model described previously, we first examined the effect of the cell density on the collective behavior of cells, with other parameters for cell–cell adhesion and contact inhibition being fixed at W_c_ = 1 and *Ψ* = 0.5, respectively. Spherical cells of radius 1 were initialized at random positions on the surface of a sphere with a radius 25, representing the YSL, and their migration simulated 500 time steps. At a cell packing density ( $\Phi =nR^{2}/4R_{E}^{2}$ for n cells on an embryo of radius $R_{E}$ ) similar to the annual killifish embryo (see Supplementary Material) (*Φ* = 0.2; 500 cells; Figure 3A and Supplementary Movie S1), the cells were able to aggregate into separated clusters and, as shown in previous studies for two-dimensional systems (Basan et al., 2013; Smeets et al., 2016), form a cohesive single aggregate at a high density (*Φ* = 0.56; 500 cells; in a smaller sphere of radius 15; Figure 3B and Supplementary Movie S2).

**FIGURE 4:**
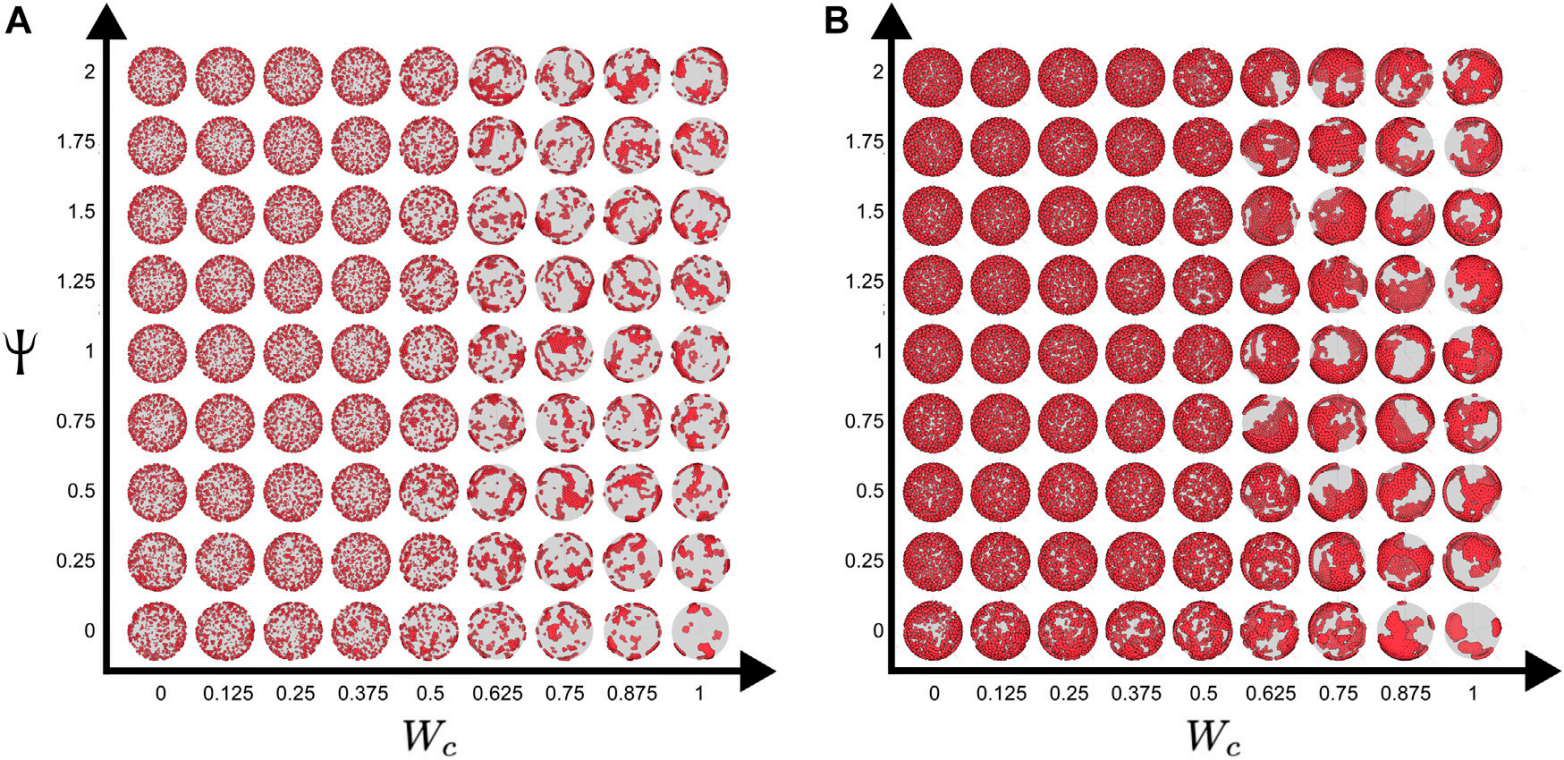
Parameter scan of the cell-autonomous system over different cell adhesion and CIL parameters. **(A)** Cell density of ϕ= 0.2 (500 cells on a sphere of radius 25). **(B)** Cell density of ϕ= 0.56 (500 cells on a sphere of radius 15). The dispersed states are maintained at low cell–cell adhesion, and clustering is increased by high cell–cell adhesion. High levels of CIL (*Ψ*) also increase cell clustering at moderate levels of cell–cell adhesion. The YSL is drawn as a transparent gray sphere; hence, the cells on the far side appear darker. The three-dimensional perspective means that these cells also appear smaller.

**FIGURE 5:**
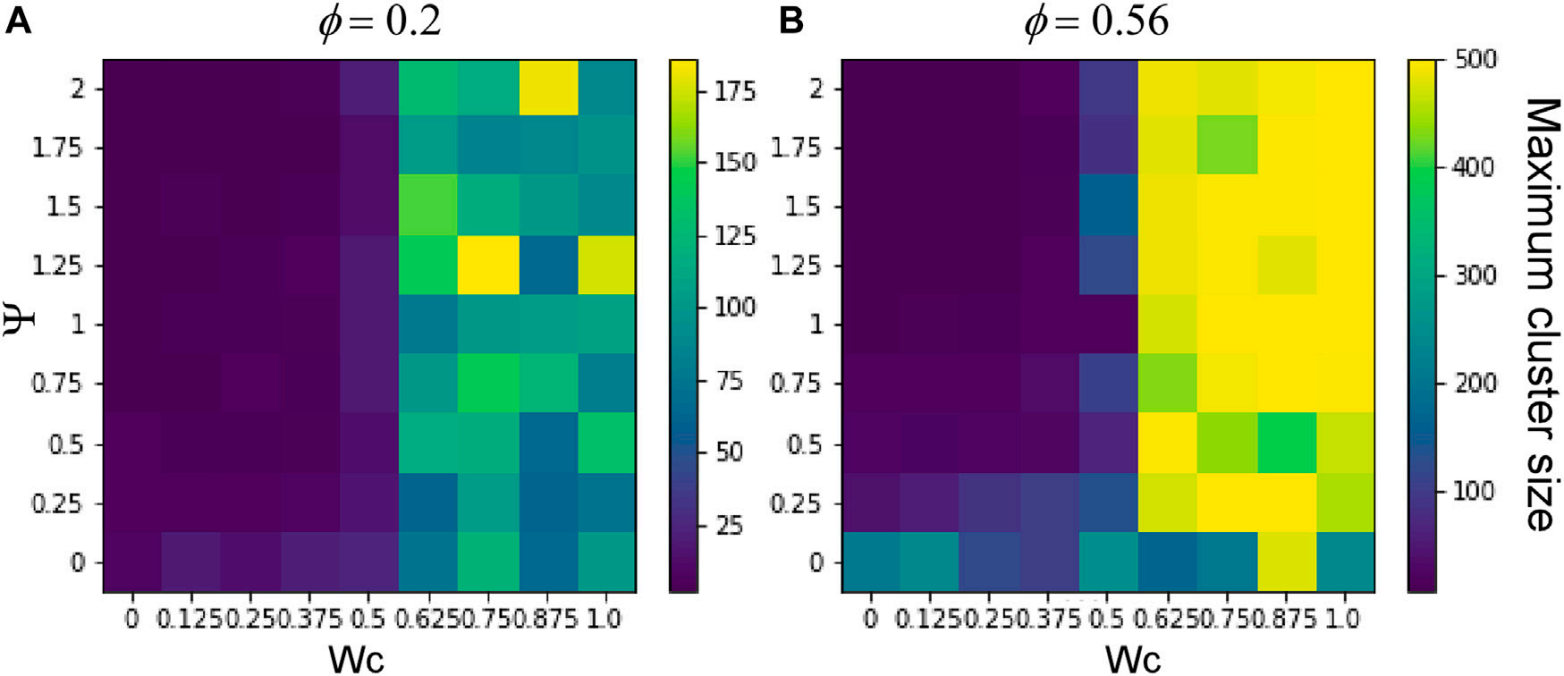
Maximum cluster size as a function of CIL and cell–cell adhesion. The maximum cluster sizes for the final states (after 500 time steps, each grid point represents the average of 25 simulations) of simulations with cell densities of **(A)** ϕ= 0.2 (500 cells on a sphere of radius 25) and **(B)** ϕ= 0.56 (500 cells on a sphere of radius 15). Clustering is increased by high cell–cell adhesion (W_c_). Both high and low levels of CIL (*Ψ*) decrease cell clustering. The plots are the averages of 25 independent simulations.

**FIGURE 6:**
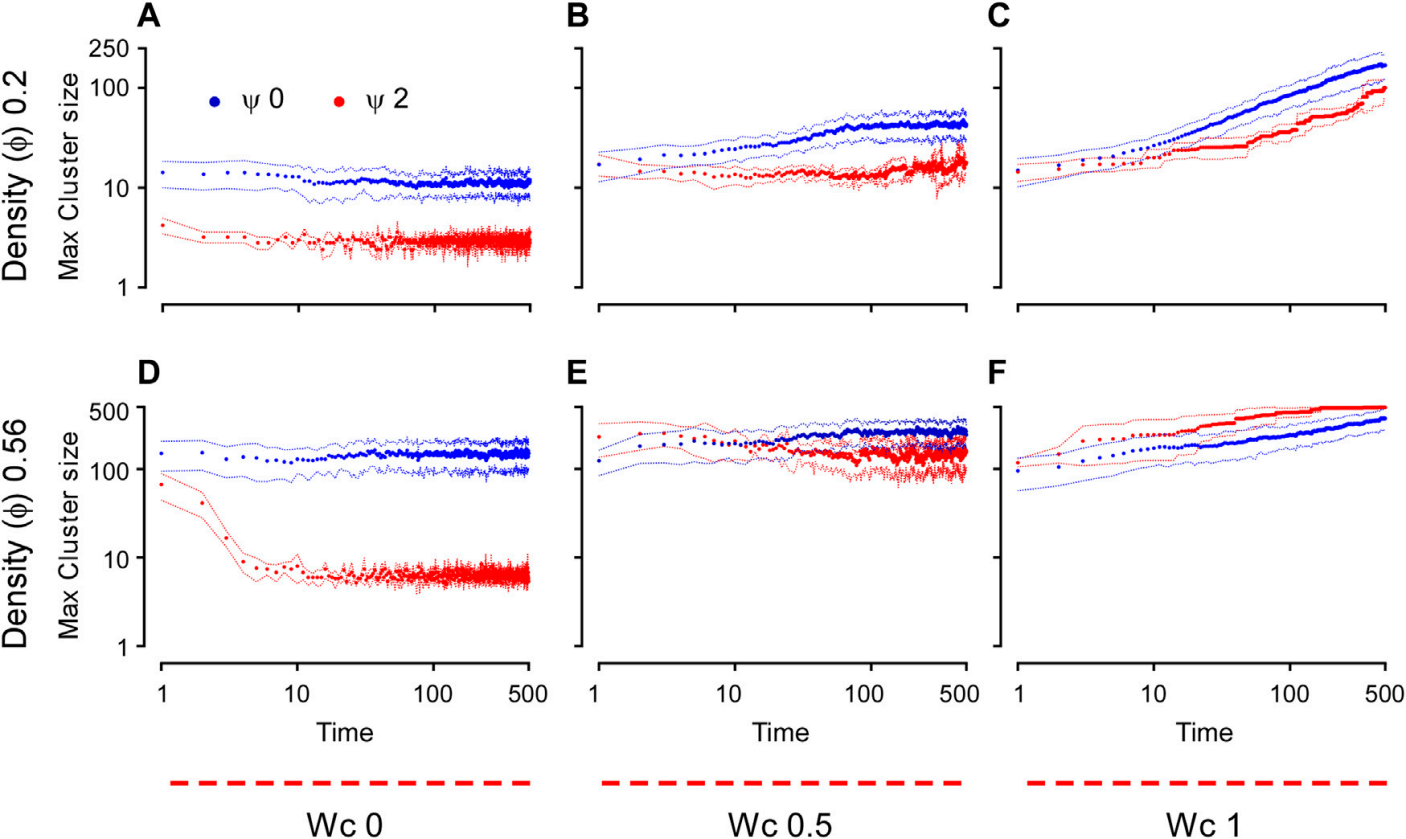
Time dynamics of the aggregation of the cell-autonomous system measured by the maximum cluster size. The cluster size is plotted here as a function of time for different cell densities, both conditions include 500 cells in each simulation ( ϕ=0.2 , **(A–C)**; ϕ=0.56 , **(D–F)** for different $W_{c}$ (0, 0.5, and 1 for the first, second, and third column, respectively) and for two different values of ψ (0 for the blue circles and 2 for the red ones). The circles are an average from 50 different simulations, and the dashed lines represent the range within the standard deviation. All plots are generated using a logarithmic scale.

To understand the effect of cell–cell adhesion and CIL on these dynamics, we scanned the parameter space, simulating the system with a range of values of W_c_ and *Ψ* for each density (*Φ* = 0.2 or 0.56). Figure 4 shows the final configurations of cells after 500 time steps for each parameter combination. A clear pattern emerges, where at low values of W_c_, the system maintains its dispersed condition, and at high W_c,_ aggregation occurs (Figures 4A, B). Similar to what was shown previously, large single aggregates can be obtained at high densities and strong cell–cell adhesions, whereas at a first approximation, *Ψ* appears to have only a marginal effect on the qualitative appearance of the cell aggregate. To quantify these effects, we computed the maximum cluster size at the end of simulations for each of the parameter combinations (Figure 5), and we also analyzed its time evolution (Figure 6). This analysis clearly confirms that larger aggregates are formed at a higher W_c_, where both cell densities are considered, and that W_c_ has a greater influence on the formation of aggregates as compared to *Ψ*. Interestingly, it also showed a sort of weak biphasic effect of *Ψ* on the cluster size, with larger aggregates being formed at intermediate values (between 0.75 and 1.5). However, high variations in final cluster sizes can be seen at these values, which may be due to the more pronounced stochasticity of the process.

Numerical simulations also allow us detailed insights into the dynamics of the aggregation process, which can be quantified by the time variation in the maximum cluster size (Figure 6). With no cell–cell adhesion (W_c_ = 0), the system was largely static, with no increase in the cluster size at either density tested after 10 dimensionless time units. This is a condition that closely resembles the dynamics observed during the dispersion state. However, as W_c_ was increased, the maximum size of the clusters tended toward the power law dynamics. On the other hand, CIL appeared to have a marginal effect on the dynamics (rate) of aggregation for intermediate and high values of W_c_ (Figures 6B, C, E, F), whereas a marked effect of CIL can be seen when W_c_ is absent (Figures 6A, D). In these conditions, a high CIL (*Ψ* = 2) caused an initial decline in the size of the cluster and a generally low aggregation as compared to *Ψ* = 0. This phenomenon, which was more pronounced at high cell densities, most likely reflects the scattering effect provided by CIL.

### 2.2 Environmental guidance

The results presented previously show that purely cell-autonomous behaviors could explain the dispersion state by keeping low cell–cell adhesion, but they were not sufficient to lead to the formation of a single aggregate at one of the embryonic poles, as seen in annual killifish early embryogenesis. It is, therefore, possible that the information provided by environmental cues is needed in the form of an organizing center that causes cells to orient toward a specific position of the embryo, where possibly a site-specific increase in W_c_ for cells reaching the location could initiate the aggregation process. The cells might preferentially move toward the organizing center by a variety of mechanisms including chemotaxis, durotaxis, and haptotaxis (CARTER, 1967; Bellomo et al., 2015; Espina et al., 2022). A powerful feature of our model and its software implementation is that such external environmental factors can easily be included. As a proof of concept, we show, here, a simple model of repolarization that shows a bias for the cells to repolarize toward the organizing center. However, experiments support a variety of possible guidance mechanisms (Sarris and Sixt, 2015a). As a feasibility study, in the Supplementary Material, we further expand two more models (i.e., “adjustment of directional speed along gradient” and “slowing down at the source”) to demonstrate that our modeling framework provides a robust and flexible tool to model a variety of taxis models (see Supplementary Material, Figures S1, S2).

#### 2.2.1 External taxis

If the organizing center is at position $x_{org}$ , the vector pointing from cell *i* to the organizing center in the tangential plane is as follows:

$$v_{i}^{org}=\left(x_{org}-x_{i}\right)-\left[\left(x_{org}-x_{i}\right)\cdot {\hat{n}}_{i}\right]{\hat{n}}_{i}.$$ (7)

Then, the angular equation of motion becomes

$${\dot{\theta }}_{i}=-f_{cil}\times \arccos\left(\frac{v_{i}^{f}∙{\hat{p}}_{i}}{\left|v_{i}^{f}\right|}\right)-f_{tax}\times \arccos\left(\frac{v_{i}^{org}∙{\hat{p}}_{i}}{\left|v_{i}^{org}\right|}\right)+\sqrt{2D_{r}}\xi$$. (8)

In this simple model, the rate of repolarization does not depend on the distance from the organizing center, nor are there any effects on the speed of cell motion. Various mechanisms and models of taxis have been proposed (Sarris and Sixt, 2015b), which while not considered here, are straightforward to implement in our modeling framework.

#### 2.2.2 Numerical simulations

**FIGURE 7:**
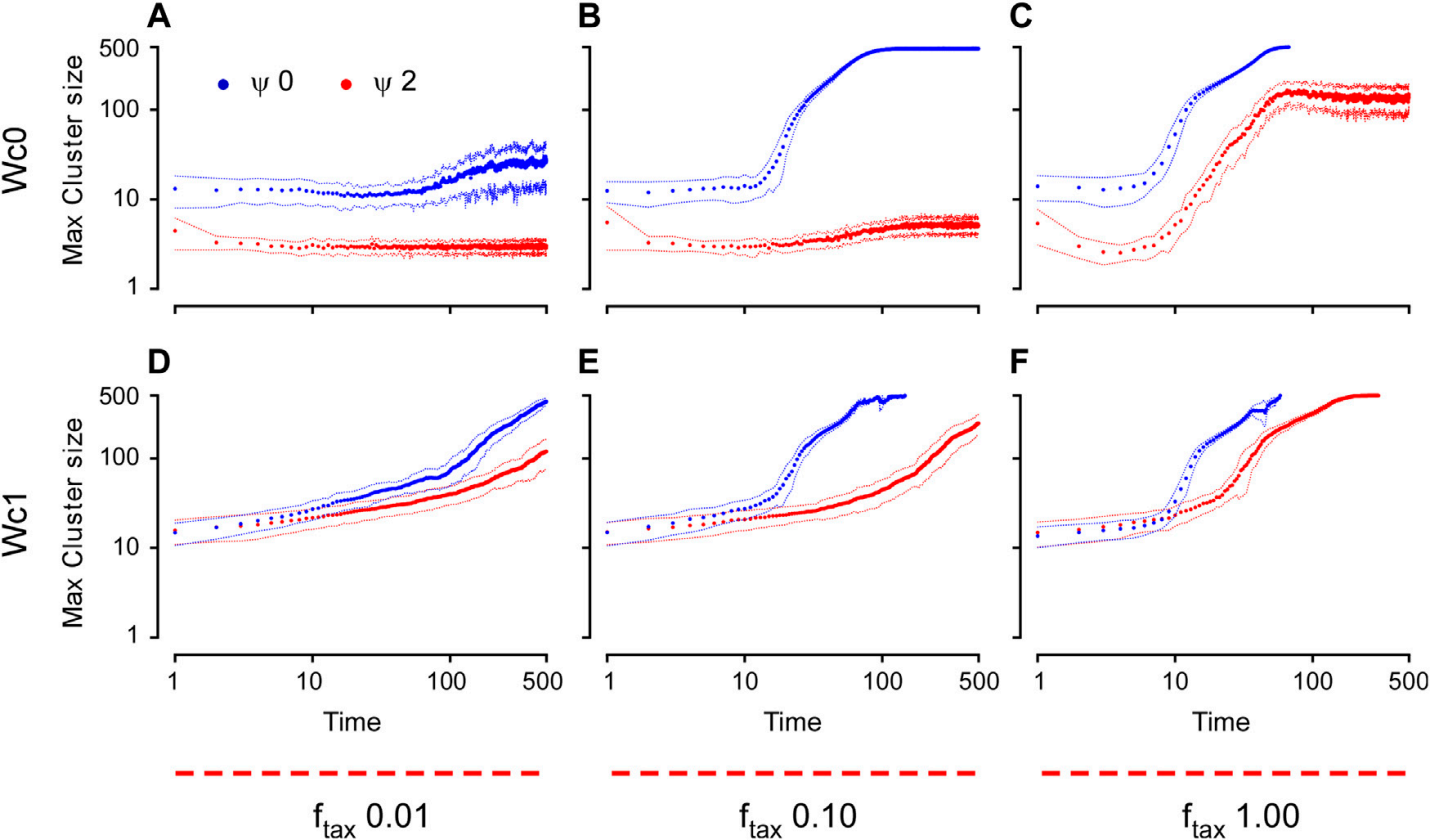
Time dynamics of aggregation for the environmental guidance model measured by the maximum cluster size. The cluster size is plotted here as a function of time for different $W_{c}$ [(0 for **(A–C)** and 1 for **(D–F)**], different *f*_*tax*_ (0.01, 0.1, and 1 for the first, second, and third column, respectively), and for two different values of ψ (0 for the blue circles, and 2 for the red ones). The data points are the average from 50 different simulations, and the dashed lines represent the standard deviation. The conditions used accurately represent the *in vivo* density of the cells ( ϕ=0.2 , 500 cells on a sphere of radius 25). All plots are generated using a logarithmic scale. The simulations were stopped when they reached a single aggregate (maximum cluster size of exactly 500 cells). Hence, the one cluster condition is only reached in those simulations that are interrupted at an earlier time [i.e., ψ = 0 in **(C, E)**; ψ = 2 in **(F)**, whereas for ψ = 0 in **(B)**, the curve gets very close but never reaches the single cluster condition].

**FIGURE 8:**
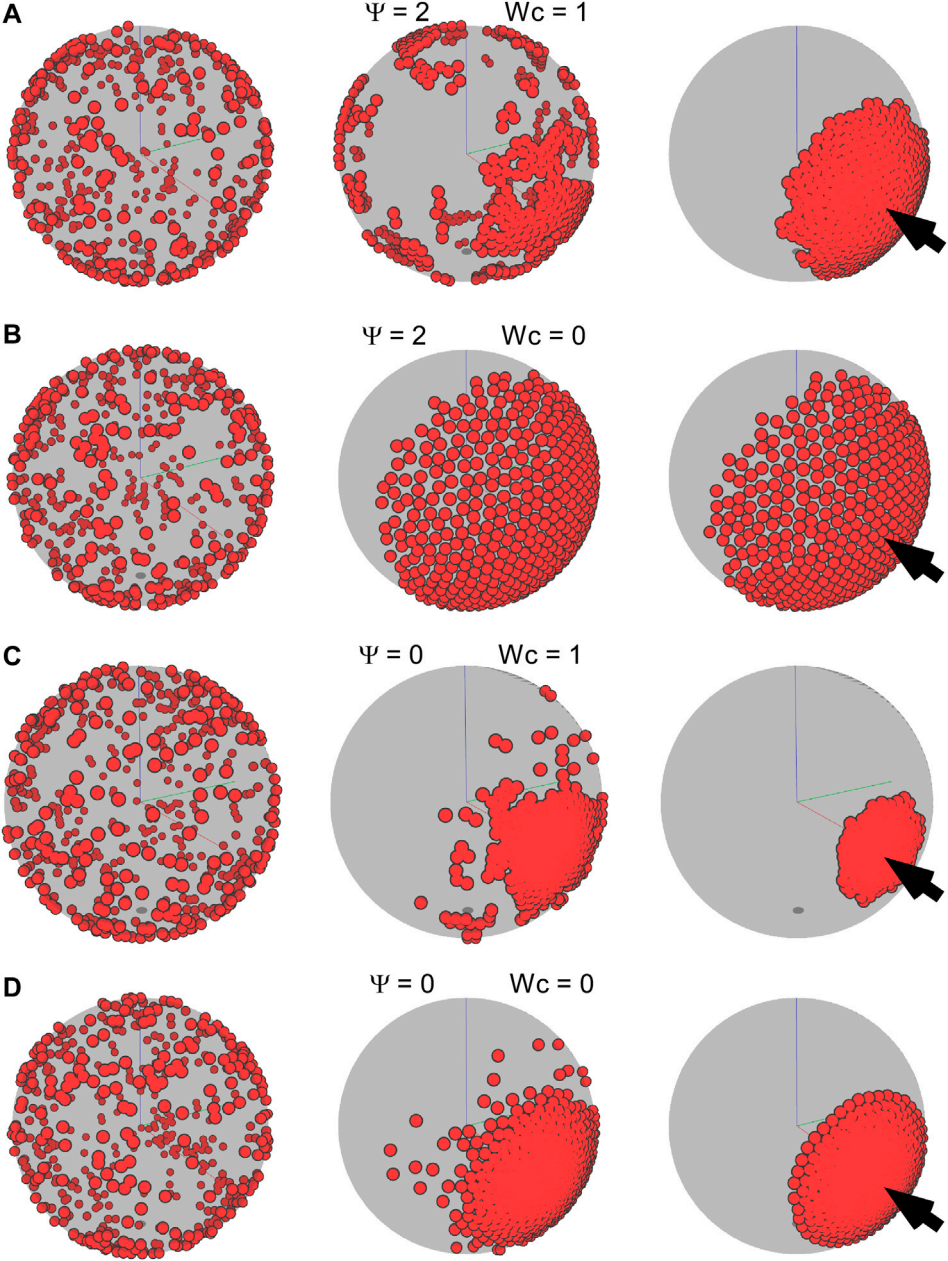
Frames of different time steps for an environmental guidance system (external taxis). For all simulations, *f*_*tax*_ is 1 and ϕ=0.2 (500 cells on a sphere of radius 25). We varied ψ and $W_{c}$ to explore the emerging aggregation dynamics and phenotypes. **(A)** ψ=2 ; $W_{c}=1.$ **(B)** ψ=2 ; $W_{c}=0.$ **(C)** ψ=0 ; $W_{c}=1.$ **(D)** ψ=0 ; $W_{c}=0.$ The YSL is drawn as a transparent gray sphere; hence, the cells on the far side appear darker. The three-dimensional perspective means that these cells also appear smaller. The images in the left, middle, and right columns represent the times 0, 50, and 500, respectively, except for C, where the final configuration is reached at time 72.5. The arrow indicates the position of the organizing center.

To test the effect of this simple external guidance (taxis), we then performed a series of simulations using the more realistic (i.e., closest to *in vivo*) conditions with cell number = 500 and sphere radius = 25, for density ϕ=0.2 , while testing the effect of varying the external taxis repolarization rate (*f*_*tax*_), cell adhesion ( $W_{c}$ ), and CIL ( ψ ). As expected, at a low *f*_*tax*_ (0.01), cells had an overall tendency to move toward the organizing center but did not form a single aggregate within the time of our simulation as the properties of the cell-autonomous system, such as rotational diffusion and CIL, prevailed (Figures 7A, D). At this low *f*_*tax*_ regime, the cells mostly remained dispersed on the surface of the sphere as single cells for $W_{c}$ = 0 (Figure 7A) or formed small aggregates that slowly coalesced at the organizing center for $W_{c}$ = 1 (Figure 7D). On the other hand, intermediate and high strengths of *f*_*tax*_ (0.1 and 1) showed features similar to the dynamics of aggregation principally depending on W_c_ and CIL, while *f*_*tax*_ determined the speed (rate) and the degree of the aggregation process with intermediate values of *f*_*tax*_ (Figures 7B, E) being, at a first approximation and to different degrees, a slower and attenuated version of the dynamics seen for the highest *f*_*tax*_ value (Figures 7C, F, 8; Supplementary Movie S3–S6). Our simulations using *f*_*tax*_ = 1 showed four distinct and equally interesting phenotypes. Strong adhesion ( $W_{c}=1$ ) combined with strong CIL ( ψ=2 ) led the cells to the formation of small aggregates that fluctuated on the surface of the sphere and eventually coalesced into a large aggregate, only at long time scales (Figure 7F, red curve; Figure 8A; Supplementary Movie S3). This was also the case for the same conditions but using *f*_*tax*_ = 0.1, where the only noticeable difference was the rate at which small aggregates moved toward the organizing center (Figure 7E, red curve). In similar conditions of CIL ( ψ=2 ), but in the absence of adhesion ( $W_{c}=0$ ), the cells moved toward the organizing center and remained in a form of dispersed single-cell dynamics within a small area close to the organizing center. However, due to high CIL, the cells did not form a tight aggregate, and the maximum cluster size plateaued at about 100 cells (Figure 7C, red curve; Figure 8B; Supplementary Movie S4). This characteristic was also seen with *f*_*tax*_ = 0.1, where the cells randomly collided with the temporarily small clusters. However, due to the low *f*_*tax*_, the cells were dispersed over a larger area as compared to *f*_*tax*_ = 1, thus diminishing the probability of collision (maximum cluster size stabilized at 3–5 cells). Finally, the aggregation into a single cluster at the organizing center was achieved when CIL was not present ( ψ=0 ), irrespective of the value of W_c_ and *f*_*tax*_ (Figures 7B, C, E, F, blue curves; Figures 8C, D; Supplementary Movie S5, S6). However, W_c_ proved to play an important role in defining the mode of migration toward the organizing center, with cells at high W_c_ moving as small aggregates (Figure 7F, blue curve; Figure 8C; Supplementary Movie S5), whereas, in the absence of adhesion (W_c_ = 0), the cells moved independently as single cells (Figure 7C, blue curve; Figure 8D; Supplementary Movie S6). Furthermore, at intermediate *f*_*tax*_ and in the absence of cell–cell adhesion (Figure 7B, blue circles), the cells at the border of the aggregate lacked the necessary pulling force from the organizing center to remain cohesively adherent to each other; thus, the aggregate approached but never reached the maximum cluster size of 500 cells. These last two conditions (Figures 7B, C, blue circles; Figure 8D; Supplementary Movie S6) seem to recapitulate the best *in vivo* observations previously reported, for example by Dolfi et al. (2014), where the cells migrate as single cells to form a loose cluster (Figure 1). However, the precise details of the migration and aggregation mechanism in annual killifish are still largely elusive. Thus, the experimental observations [as in (Dolfi et al., 2014)] need to be corroborated, expanded, and carefully analyzed before being implemented in our model.

## 3 Discussion

We have developed a computational model of cell migration and aggregation mechanisms for cells trapped between two concentric spheres, as is the case of the *in vivo* conditions of the annual killifish early embryo development. We demonstrate that numerical simulations of this model are an essential tool to test the role of physical mechanisms based on both the cell-autonomous and environmental guidance factors in driving the complex self-organizing processes during morphogenesis. Our computational model includes the full geometry of the embryo, including the spherical topology due to the constraint between the EVL and the YSL. This model was solved numerically to build phase space maps that reveal the effect of key mechanical parameters on the aggregation process.

For cell-autonomous mechanisms, the simulated aggregate phenotype was comparable to the experimental observation of the dispersed state, which could be achieved simply if the cells had low adhesion but failed to recapitulate the aggregation into a single cluster (Figures 3–6). At high adhesion, smaller clusters fused into a larger cluster, but the type of coalescence observed *in vivo* was never observed under these simple conditions. Thus, we explored the possibility that in addition to cell-autonomous factors, external cues possibly arising from a putative organizing center are also at play. The environmental taxis cues that guide cell migration could, in principle, be of many kinds, possibly chemical (i.e., diffusible or substrate-bound gradients of a chemoattractant) or physical (i.e., stiffness gradients). Using our model, we simulated a simple external cue guiding the direction of the migration of cells and showed that, under some conditions, it does indeed form single localized clusters (Figures 7, 8). In particular, our simulations determined that in order to achieve the dynamic and resulting phenotype of aggregation observed in early killifish development, an organizing center and low levels of CIL are essential. In addition, it appears that adhesive forces must remain low throughout the process and possibly increase only after the aggregate has formed.

Importantly, our strategy aims at building knowledge using a bottom-up approach by making predictions starting from the minimal condition for aggregation. Thus, at present, our model is kept to the most simplistic conditions possible in order to avoid overfitting of the system. However, more detailed models of cell motility or taxis, for example, may easily be constructed using our framework, given the relevant mechanistic details and more complete experimental evidence. Another aspect that might be considered is lateral inhibition, a mechanism that implicates the cell inhibition of adjacent cell activity. We are currently exploring all these mechanisms to simulate an aggregation process that is most like the experimental phenomena seen in annual killifish. Most importantly, despite its simplicity, our model is able to recapitulate the major features of cell aggregation in early annual killifish embryo development, a nearly unique model of cell aggregation in vertebrates.

## 4 Methods

### 4.1 Numerical simulations

The equations of motion (1) were solved using an implicit Euler method, with

$$x_{i}(t+\Delta t)-x_{i}(t)=\frac{\Delta t}{\gamma_{s}}(-F_{m}{\hat{p}}_{i}+\underset{j}{\sum }F_{ij}^{cc}\frac{\left(x_{i}\left(t+\Delta t\right)-x_{j}\left(t+\Delta t\right)\right)}{\left|x_{i}\left(t+\Delta t\right)-x_{j}\left(t+\Delta t\right)\right|})$$ (9)

so that we solve for $x_{i}\left(t+\Delta t\right)$ implicitly from Eq. 9. The rotational equations of motion given by Eqs 6, 8 were solved using the Euler–Maruyama scheme, such that

$$\theta_{i}\left(t+\Delta t\right)=\theta_{i}\left(t\right)+\Delta t\left[f_{pol}\left(\theta_{i}^{*}\left(t\right)-\theta_{i}\left(t\right)\right)\right]+\sqrt{2D_{r}}\xi_{i},$$ (10)

where $\xi_{i}$ is the normally distributed zero mean random noise with variance Δt . In all simulations, Δt was chosen as 0.1 for cell-autonomous and external taxis models.

For each set of parameters, 50 replicates were made for cell-autonomous and 50 for external taxis models.

### 4.2 Software implementation

All codes and models were implemented using the open-source CellModeller multicellular modeling framework (Rudge et al., 2012).

### 4.3 Embryo imaging

The *in vivo* experiments depicted in Figure 1 were performed under the license for animal housing, breeding, and manipulation that was issued by Umwelt-und Verbraucherschutzamt der Stadt Köln, with the authorization no. 576.1.36.6.G28/13 Be. All the fish used were raised in 35-L tanks at 24°C–26°C and belonged to the *N. furzeri* FUCCI transgenic strain published by Dolfi et al. (2019). They were fed two to three times a day with frozen Chironomus larvae or living nauplii of *Artemia salina*, depending on their size. The breeders were kept in 8-L tanks with one or more boxes (9 cm × 9 cm × 4 cm) filled with 2 cm of river sand and were left to spawn eggs for 1 h. The embryos were collected by sieving the sand with a plastic net and were then embedded in 2% low melting agar. The fluorescence images shown in Figure 1 were acquired using a Leica TCS SP5-X confocal microscope and the red emission channel with 543-nm lasers. A total of 40 to 60 images per embryo were acquired at a depth distance of 6 μm. The maximum intensity projections shown have been generated using ImageJ. The contrast in the images has been adjusted, but not altered, to optimize visualization.

## Data Availability

The raw data supporting the conclusion of this article will be made available by the authors upon request.
