# Supplementary material for: A computational framework for testing hypotheses of the minimal mechanical requirements for cell aggregation using early annual killifish embryogenesis as a model
Source: Front Cell Dev Biol. 2023 Mar 20;11:959611. doi: 10.3389/fcell.2023.959611 (PMC10067630; doi:10.3389/fcell.2023.959611)
Supplement: Supplementary file 2 [file DataSheet1.PDF]

Supplementary information: A computational framework for testing hypotheses of the minimal mechanical requirements for cell aggregation using early annual killifish embryogenesis as a model

Density estimation for annual killifish embryo

Based on our microscopy data we counted the cell number as n=324, the embryo radius as R_E=628μm and the cell radius as R=33μm. This gives an embryo radius in dimensionless units of cell radii as 20, and a cell density of $\Phi = n\, R^2/4\, R_E^2 = 0.22$.

Stability of implicit Euler integration scheme

Consider two cells of radius R moving in opposite directions along the horizontal x-axis with positions x_1 and x_2 ($x_1 < x_2$), colliding such that $x_2 - x_1 < 2R$, we have from equations 1-3 of the main text:

$$\gamma_s \dot{x}_1 = F_m - \frac{2}{R}\left[W_s - \frac{W_s + W_c}{R}(\mathrm{x}_2 - x_1 - R)\right]$$

$$\gamma_s \dot{x}_2 = -F_m + \frac{2}{R}\left[W_s - \frac{W_s + W_c}{R}(\mathrm{x}_2 - x_1 - R)\right]$$

Now let $y = x_2 - x_1$ then,

$$\dot{y} = -2\frac{F_m}{\gamma_s} + \frac{4}{\gamma_s R}\left[W_s - \frac{W_s + W_c}{R}(y - R)\right] = -ay + b$$

where

$$a = \frac{4}{\gamma_s R^2}(W_s + W_c)$$

and

$$b = -2\frac{F_m}{\gamma_s} + \frac{4}{\gamma_s R}(2W_s + W_c)$$

Now let $z = ay - b$, then

$$\dot{z} = a\dot{y} = -az$$

It can be shown (*1*) that the implicit Euler numerical integration scheme for this differential equation is stable when $a \geq 0$ so that in this case our numerical method is stable when

$$\frac{4}{\gamma_s R^2}(W_s + W_c) > 0$$

Since $R > 0$, $\gamma_s > 0$, $W_s > 0$, and $W_c > 0$, our method is always stable for two colliding cells.

Limit on time step due to collision detection

Now consider two non-colliding cells similarly moving along the x-axis, we now have,

$$\gamma_s \dot{x_1} = F_m$$

$$\gamma_s \dot{x_2} = -F_m$$

Thus, integrating numerically, we obtain,

$$x_1(t + \Delta t) = x_1(t) + \frac{F_m}{\gamma_s}\Delta t$$

$$x_2(t + \Delta t) = x_2(t) - \frac{F_m}{\gamma_s}\Delta t$$

To correctly detect collisions between the cells, the distance between them $y = x_2 - x_1$ should not become less than R after a time step of Δt. We have,

$$y(t + \Delta t) = y(t) - \frac{2F_m}{\gamma_s}\Delta t > R$$

for all $y(t) > 0$. Since the cells are not colliding, the minimum possible value of $y(t)$ is $2R$ and so,

$$2R - \frac{2F_m}{\gamma_s}\Delta t > R$$

and thus

$$\Delta t < \frac{\gamma_s R}{2F_m}$$

With the parameters used in this work ($F_m = 1, R = 1, \gamma_s = 1$), we have that $\Delta t < 1/2$. We used $\Delta t = 0.1$ for all our simulations in the manuscript.

Limit on time step due to repolarization model

Considering the repolarization model given in main text equation 5, Smeets *et al.*(*2*) state that the upper limit on the time step Δt is such that $\Delta t < 1/f_{pol}$, where f_{pol} is the repolarization rate. In our case the maximum value of f_{pol} is 4 ($f_{pol} = 2D_R\psi$, $D_R = 1$,) so that we must maintain $\Delta t < 1/4$.

Other proposed models of cell taxis

Adjustment of directional speed along gradient

In this model the motile force of cells is larger when their polarity is aligned with the direction of the organizing center (gradient). We model this as,

$$F_i = 1 + p_i \cdot v_i^{org} \quad (1)$$

where F_i is the motile force, p_i is the polarity vector, and v_i^{org} (see equation 7 of main text) is the direction of the organizing center for cell i. Sup. Figure 2 shows the resulting clustering dynamics of three simulations with and without cell-cell adhesion, showing a clear tendency to form a single large cluster even in the absence of adhesion between cells.

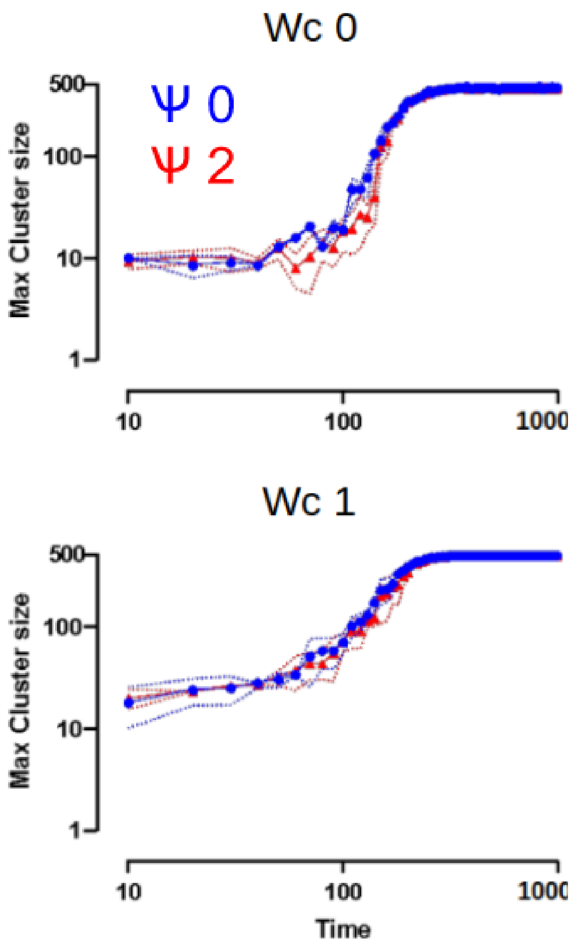

Suppl. Figure 1. Time dynamics of aggregation of a different taxis model (adjustment of directional speed along gradient) measured by maximum cluster size. Cluster size is here plotted as a function of time for different W_c (0 for the top row and 1 for the bottom row) and for two different values of ψ (0 for the blue circles and 2 for the red). Circles are the average from 3 different simulations and dashed lines represent the standard deviation. Plots are made using a log-log scale for both axes.

Slowing down at the source

In this model cells move more slowly as they approach the organizing center. We model this as,

$$F_i = 1 - exp\left(-\frac{D_i}{\xi}\right) \text{ (2)},$$

where F_i is the motile force and D_i is the distance to the organizing center for cell *i.* Sup. Figure 3 shows the clustering dynamics for three simulations with and without cell-cell adhesion, and with $\xi = 10$ dimensionless units. It is clear that minimal clustering occurs except in the presence of cell-cell adhesion.

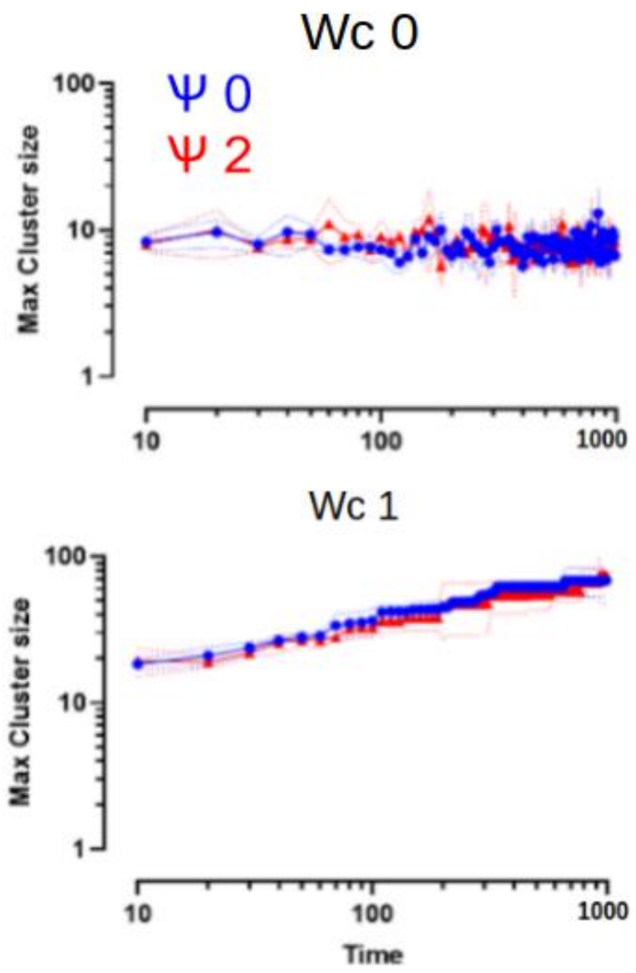

Suppl. Figure 2. Time dynamics of aggregation of a different taxis model (slowing down at the source) measured by maximum cluster size. Cluster size is here plotted as a function of time for different W_c (0 for the top row and 1 for the bottom row) and for two different values of ψ (0 for the blue circles and 2 for the red). Circles are the average from 3 different simulations and dashed lines represent the standard deviation. Plots are made using a log-log scale for both axes.

References

1. W. H. Press, S. A. Teukolsky, W. T. Vettering, B. P. Flannery, *NUMERICAL RECIPES The Art of Scientific Computing Third Edition* (2007).
2. B. Smeets *et al.*, *Proc Natl Acad Sci U S A*. **113** (2016), doi:10.1073/pnas.1521151113.
